# A novel protein encoded by circIMP3 promotes prostate cancer progression by regulating alternative splicing and tumor microenvironment

**DOI:** 10.3389/fcell.2025.1722674

**Published:** 2026-01-05

**Authors:** Wenren Zuo, Weizhou Huang, Haojie Chen, Yan Xu, Yang Zhang

**Affiliations:** 1 Department of urology, Affiliated Hospital of Nanjing University of Chinese Medicine, Nanjing, China; 2 Graduate School of Nanjing University of Chinese Medicine, Nanjing, China

**Keywords:** prostate cancer, circular RNA, alternative splicing, IMP3, Fbxw7, c-Myc

## Abstract

**Introduction:**

Prostate cancer (PC) is one of the most prevalent malignancies in men, with rising incidence and mortality rates globally. Despite advances in therapeutic options such as androgen deprivation therapy and chemotherapy, effective cures, especially for advanced stages of the disease, remain limited. Recent research has highlighted the significant roles of alternative splicing (AS) and noncoding RNAs in tumor progression and drug resistance. This study aims to investigate the role of circIMP3, derived from the IMP3 gene, in prostate cancer development.

**Methods:**

In this study, we employed quantitative PCR, RNA sequencing, and immunoblotting to identify and characterize circIMP3 in prostate cancer tissues and patient blood samples. Functional assays, including cell proliferation and *in vivo* tumorigenicity assays, were conducted to assess the biological role of circIMP3 in PC cells. RNA immunoprecipitation sequencing (RIP-seq) was used to identify alternative splicing events regulated by circIMP3. Additionally, exosome isolation and uptake assays were performed to explore the paracrine signaling function of circIMP3 within the tumor microenvironment (TME).

**Results:**

We identified circIMP3, which is significantly upregulated in both prostate cancer tissues and peripheral blood of patients. CircIMP3 contains an internal ribosome entry site (IRES) and encodes a previously uncharacterized 288-amino-acid protein, circIMP3_288aa. Functional assays revealed that circIMP3_288aa promotes cell proliferation *in vitro* and accelerates tumor growth *in vivo*. Mechanistically, circIMP3_ 288aa regulates the alternative splicing of FBXW7, leading to impaired c-Myc ubiquitination and stabilization, which enhances oncogenic signaling. RIP-seq analysis identified over 2,000 alternative splicing events regulated by IMP3, with a notable enrichment in pathways related to ubiquitin-mediated proteolysis. Furthermore, circIMP3 is secreted into the TME via exosomes, where it is taken up by recipient cells, contributing to their proliferation.

**Discussion:**

Our findings demonstrate that circIMP3 acts as a key regulator of both intracellular alternative splicing and extracellular paracrine signaling within the TME. The ability of circIMP3 to influence FBXW7 splicing and stabilize c-Myc provides a mechanistic basis for its role in promoting oncogenesis in prostate cancer. Clinically, high expression levels of circIMP3 correlate with poorer event-free survival in prostate cancer patients, suggesting its potential as a prognostic biomarker. Additionally, the detection of circIMP3 in peripheral blood positions it as a promising target for liquid biopsy applications in PC diagnosis and monitoring.

## 1 Introduction

Prostate cancer (PC) is a malignant tumor originating from the epithelial cells of the prostate and is closely linked to alternative splicing (Cornford et al., 2024). It ranks among the most prevalent cancers in men. According to the 2023 U.S. cancer statistics, prostate cancer accounts for over one-quarter of all new male cancer cases and is the second leading cause of cancer-related deaths (Tilki et al., 2024). From 2014 to 2019, its incidence increased by approximately 3% annually, with an estimated 288,300 new cases and 34,700 deaths projected for 2025 (Kumar et al., 2024). In China, the incidence of PC has been rising steadily, driven by an aging population and changing dietary patterns (Liu et al., 2024; Wilson and Zishiri, 2024). In 2022, approximately 125,646 new cases and 56,239 deaths were reported, with both figures continuing to climb (Rajadnya et al., 2024).

Current treatments include androgen deprivation therapy, anti-androgen therapy, chemotherapy, and emerging approaches such as CAR-T therapy (Bakht and Beltran, 2025; Chou et al., 2025). However, a definitive cure for PC remains elusive. As the aging trend intensifies, the need for novel therapeutic targets becomes increasingly urgent. Recent advances in understanding the interactions between tumor cells and their surrounding microenvironment have significantly contributed to the development of novel therapeutic strategies for PC (Luo and Wen, 2024; Palecki et al., 2024). Among the various mechanisms of intercellular communication within the tumor microenvironment, exosomes and circular RNAs (circRNAs) have emerged as important mediators attracting growing interest (Conn et al., 2024; Kohansal et al., 2024). CircRNAs are a unique class of covalently closed, single-stranded RNA molecules generated through back-splicing of exonic or intronic sequences from precursor mRNAs (Zhang et al., 2024). These molecules are highly stable, evolutionarily conserved, and abundant in both exosomes and body fluids, which suggests a potential role in cell-to-cell communication (Yang and Li, 2024; Liang et al., 2024). Accumulating evidence indicates that circRNAs play crucial roles in cancer development, progression, and resistance to therapy. In the context of prostate cancer, circRNAs have been implicated in modulating androgen receptor signaling, regulating oncogenes and tumor suppressors, and reshaping the tumor microenvironment (Hu et al., 2024). Moreover, the presence of circRNAs in extracellular vesicles enables them to participate in intercellular crosstalk, potentially influencing immune evasion, stromal remodeling, and metastatic spread (Shi et al., 2024; Mohanapriya et al., 2022). Due to their stability and detectability in liquid biopsies, circRNAs are now being explored as promising diagnostic and prognostic biomarkers for PC (Ding et al., 2024; Li H. et al., 2024).

The recent progress in circRNA research has prompted us to investigate whether the malignancy of PC cells is, at least in part, driven by circRNA-mediated mechanisms. In our study, we identified a specific circRNA, circIMP3, as being highly enriched in PC patient tissues. Further analysis revealed that circIMP3 encodes a previously uncharacterized 288-amino acid protein, which we named circIMP3_288aa.Intriguingly, circIMP3 was found to be actively secreted into the tumor microenvironment via exosomes, raising the possibility that it may participate in modulating intercellular communication and promoting tumor progression. Both *in vitro* and *in vivo* models were used to explore the functional roles of circIMP3 and its protein product in PC. Our data demonstrated that circIMP3_288aa promotes proliferation through regulating FBXW7 alternative splicing. Moreover, we identified a novel downstream effector of circIMP3_288aa, suggesting a previously unknown signaling axis in prostate cancer biology. These findings provide important insights into the oncogenic potential of translatable circRNAs in PC and highlight circIMP3_288aa as a promising prognostic biomarker and potential therapeutic target.

## 2 Materials and methods

### 2.1 Antibodies and reagents

The primary antibodies were IMP3 (2774s, Cell Signaling Technology), FLAG (9661S, Cell Signaling Technology), HA (51064–2 AP, ProteinTech Group, China), β actin (4970S, Cell Signaling Technology, United States). The second antibodies included goat anti Rabbit IgG (H + L) HRP (FMS Rb01, Fcmacs) or mouse (S0002, Affinity) were at the dilutions of 1:5000. The goat anti rabbit IgG/Alexa fuor 647 (BS 0295 G, Bioss, China), Goat pab to Ms IgG (FITC) (ab6785, abcam, United Kingdom) were at the 1:200 dilution.

Trizol reagent (10606ES60), Hifair^1st^ Strand cDNA Synthesis SuperMix for qPCR (gDNA digesterplus) (11121ES60) and SYBR Green PCR master mix (11201ES03) were purchased from Yeasen Biotechnology (Shanghai) Co., Ltd.

### 2.2 Cell lines and cell culture

LNCaP and PC3 cells were cultured in RPMI 1640 (Biological Industries, Israel). RPMI 1640 was supplemented with 10% fetal bovine serum (Gibco, United States), 100 U/mL penicillin, and 100 μg/mL streptomycin (HyClone, United States). The cells were cultured at 37 °C in 5% CO_2_.

### 2.3 Plasmids and transfection

CircRNA generation was conducted as previously described (Tang et al., 2022). The circRNADb database (http://reprod.njmu.edu.cn/cgi-bin/circrnadb/circRNADb.php) was adopted to predict the open reading frame (ORF) of circIMP3. A commercially available circRNA expression vector PLC5-ciR (GS0104, Guangzhou Geneseed Biotech Co, China) was used to construct a circIMP3-overexpression (OE) vector according to the predicted translation mode. To induce circularization *in vivo*, side flanking repeat sequences and SA/SD sequences were added to both sides of the 638 nt sequences (OV-circIMP3). The front circular frame contained the endogenous flanking genomic sequences with EcoRI restriction enzyme site, and the back-circular frame included part of the inverted upstream sequence with BamHI site. Cancer cells were transfected using lentivirus, as described previously.

### 2.4 Cell proliferation, colony formation, and cell apoptosis assays

Cell proliferation, colony formation, and apoptosis were assessed using CCK8, soft agar colony formation, and Annexin V/PI staining, as previously described (Tang et al., 2022).

### 2.5 Transfection and preparation of EVs

HEK293 cells were co-transfected with circIMP3 lentivirus and Lamp2b lentivirus to generate HEK293 circIMP3-OE cells, respectively. After 48 h of transfection, puromycin selection was used to isolate stably transfected cells. Exosomes containing circIMP3 (circIMP3-EVs) were isolated from the supernatant of HEK293 Lamp2b circIMP3-OE and HEK293 circIMP3-OE cells. We purified circIMP3-EVs using a series of centrifugation steps. First, we spun the samples at 300×g to remove cells. Next, we increased the speed to 10,000×g to remove shedding vesicles. Finally, we spun the samples at 3,000×g to remove any remaining cell debris. The supernatant was filtered through a 0.22 μm filter and subjected to ultracentrifugation at 200,000×g for 90 min. The resulting pellets were resuspended in PBS and purified through additional ultracentrifugation at 200,000×g.

### 2.6 Characterization of EVs

Western blotting (WB) was used to analyze the expression of Alix and CD9, two marker proteins of exosomes, in the purified EVs. The morphology of the EVs was characterized using a JEOL 2100 transmission electron microscope. Nanoparticle tracking analysis (NTA) was employed to determine the size distribution of the particles.

### 2.7 Establishment of the subcutaneous xenograft model

WT and circIMP3-OE PC cells (1 × 10^6^) were subcutaneously injected into the bilateral flanks of 6–8-week-old nude mice. Tumor growth was monitored every 2 days, and tumor volume was calculated according to the formula: length × width^2^/2. Tumors were harvested for photographing and weighed once they had reached a diameter of 15 mm.

### 2.8 RNA immunoprecipitation sequencing (RIP-seq)

RNA immunoprecipitation (RIP) was carried out following established protocols with slight modifications (Vaňková Hausnerová et al., 2024). A total of 5–20 × 10^6^ cells were harvested and lysed to extract proteins. Magnetic Protein A/G beads were pre-incubated with 5 μg of anti-HA antibody, followed by incubation with the cleared cell lysates to capture RNA–protein complexes. After immunoprecipitation, the bead-bound complexes were treated with 150 μL of proteinase K digestion buffer to release the bound RNAs. The enriched RNAs were subsequently purified and subjected to downstream analysis, including RT-qPCR or high-throughput sequencing. RIP-seq was conducted using the Illumina sequencing platform provided by Novogene Co., Ltd. (Beijing, China).

### 2.9 Identification of AS events

Alternative splicing (AS) events from RIP-seq datasets were analyzed using rMATS (version 4.1.0). Splicing variants were detected and quantified based on read alignment, and statistically significant AS events were defined by thresholds of *P* < 0.05 and |IncLevelDifference| > 0.05. Representative AS patterns were visualized using Sashimi plots. For Nanopore long-read sequencing data, AS events and splice isoform abundances were identified using FLAIR (version 1.5) with the following parameters: diffSplice-drim1 3-drim2 three test. Differential AS events between groups were subsequently evaluated using DRIMSeq, and events with *P* < 0.05 were considered significant. Gene expression profiles were further quantified using Cufflinks, and log2 fold changes were incorporated into cumulative distribution analyses. Cumulative distributions were calculated using the ddply package, and statistical significance between conditions was determined by pairwise Wilcoxon tests with Bonferroni adjustment. Data visualization, including cumulative distribution plots, was performed using the ggplot2 package in R.

### 2.10 Mass spectrometry analysis

Proteins were resolved using SDS-PAGE, and gel slices corresponding to the target molecular weight were carefully excised and subjected to enzymatic digestion. The resulting peptide mixtures were analyzed using a Q Exactive mass spectrometer (Thermo Fisher Scientific). Acquired fragmentation spectra were interpreted based on the NCBI non-redundant protein database for protein identification.

### 2.11 RNase R treatment

To selectively remove linear RNAs, RNase R (Epicentre Biotechnologies, Madison, WI, United States) treatment was performed. Total RNA was isolated from ARP1 and CAG cells and then equally divided into two aliquots: one subjected to RNase R digestion and the other incubated with digestion buffer alone as a negative control. For the treatment group, 20 μg of total RNA was incubated with RNase R (20 U/μL) at 37 °C for 15 min.

### 2.12 Statistical analyses

Statistical analyses were performed using SPSS version 22.0, and all values were expressed as mean ± standard deviation (SD) unless otherwise specified. A two-tailed Student’s t-test (2 groups) or one-way analysis of variance (ANOVA) (≥3 groups) was utilized to evaluate statistical significance. *P* < 0.05 was considered statistically significant.

## 3 Results

### 3.1 High IMP3 expression indicates poor clinical outcome and increased tumor growth in prostate cancer

**FIGURE 1:**
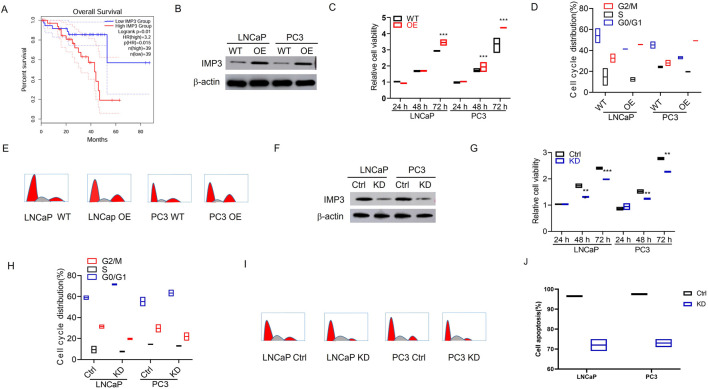
High IMP3 expression indicates poor clinical outcome and promotes tumor growth in prostate cancer. **(A)** Elevated IMP3 expression was associated with poorer event-free survival (EFS). **(B)** Western blot analysis confirmed stable overexpression of IMP3 in LNCaP and PC3 cells. **(C)** CCK-8 assays showed that IMP3 overexpression significantly enhanced the proliferation of LNCaP and PC3 cells (p < 0.001). **(D,E)** Flow cytometry analysis demonstrated an increased proportion of cells in the G2/M phase in IMP3-overexpressing cells relative to wild-type controls (p < 0.05). **(F)** Western blot analysis confirmed efficient knockdown of IMP3 in LNCaP and PC3 cells using lentiviral shRNA. **(G)** Cell proliferation was markedly reduced following IMP3 knockdown (p < 0.001). **(H,I)** IMP3 silencing led to a significant decrease in the G2/M phase cell population (p < 0.01). **(J)** Annexin V/PI staining revealed that IMP3 knockdown induced apoptosis in PC cells.

The TCGA cohorts were analyzed to evaluate the association between IMP3 expression and overall survival in PC. The results showed that high IMP3 expression was correlated with poorer clinical outcomes (*P* < 0.05) (Figure 1A). We further explored the functions of IMP3 in PC cell lines. Initially, we stably overexpressed IMP3 in LNCaP and PC3 cells confirmed by WB analysis (Figure 1B). The proliferation capacity of LNCaP and PC3 cells was remarkably increased upon upregulating IMP3 expression (p < 0.001) (Figure 1C). Cell cycle analysis demonstrated an evidently higher G2/M phase proportion in IMP3-OE cells than WT cells (p < 0.05) (Figures 1D,E). Conversely, we transfected PC cells with IMP3 lentiviral shRNA particles, and the knockdown efficiency was validated by WB analysis (Figure 1F). The cell proliferation was significantly decreased in IMP3-KD cells compared with control cells (p < 0.001) (Figure 1G). IMP3-KD PC cells resulted in a significant decrease in the G2/M phase proportion (p < 0.01) (Figures 1H,I). Consistent with these findings, AV/PI staining confirmed that IMP3 KD induced cell apoptosis Figure 1J). Taken together, these observations further support the notion that IMP3 stimulates PC cell growth.

### 3.2 Elevated IMP3 induces drug resistance in PC

**FIGURE 2:**
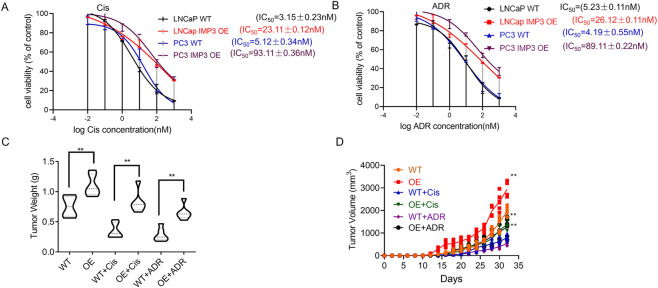
IMP3 overexpression promotes chemoresistance to cisplatin and doxorubicin in prostate cancer. **(A)** MTT assays revealed that IMP3 overexpression significantly increased cisplatin resistance in LNCaP and PC3 cells. **(B)** Similarly, IMP3-OE cells exhibited increased resistance to doxorubicin (ADR). **(C,D)** *In vivo* xenograft models using NOD-SCID mice showed that tumors derived from IMP3-overexpressing LNCaP cells grew significantly larger than those from WT cells following ADR or Cis treatment.

To further explore the relationship between IMP3 expression and chemoresistance, we performed MTT assays to evaluate the response of IMP3 WT and OE cells to cisplatin (Cis) and doxorubicin (ADR). IMP3 overexpression resulted in a marked increase in cisplatin IC50 values in both LNCaP and PC3 cell lines. Specifically, the IC50 of cisplatin in LNCaP IMP3-OE cells was (23.11 ± 0.12) nM, in contrast to (3.15 ± 0.23) nM in LNCaP WT cells. Similarly, PC3 IMP3-OE cells showed an IC50 of (93.11 ± 0.36) nM, compared to (5.12 ± 0.34) nM in PC3 WT cells, indicating that elevated IMP3 expression enhances cisplatin resistance (Figure 2A). A comparable trend was observed with ADR treatment. The IC50 in LNCaP IMP3-OE cells increased to (26.12 ± 0.11) nM, while the WT counterpart remained at (5.23 ± 0.11) nM. In PC3 cells, IMP3 overexpression raised the IC50 to (89.11 ± 0.22) nM from (4.19 ± 0.55) nM in WT cells, further supporting the role of IMP3 in promoting resistance to doxorubicin (Figure 2B). To assess these findings in an *in vivo* context, LNCaP cells with either WT or OE IMP3 were subcutaneously implanted into the flanks of NOD-SCID mice, followed by administration of Adriamycin (ADR) or Cisplatin (Cis). After 32 days, tumors derived from IMP3-OE cells demonstrated significantly accelerated growth, with greater average weight and volume than those from WT cells (Figures 2C,D). Cis treatment reduced tumor volume by 70% in the WT group, but only by 30% in the OE group, indicating reduced sensitivity. A similar trend was observed with ADR treatment. While WT tumors responded to both ADR and Cis with decreased growth, tumors from IMP3-OE cells showed no notable change in size following either treatment. These results indicate that IMP3 overexpression confers resistance to ADR and Cis *in vivo*, in contrast to the chemosensitivity seen in WT tumors.

### 3.3 Identification of IMP3-regulated alternative splicing events

**FIGURE 3:**
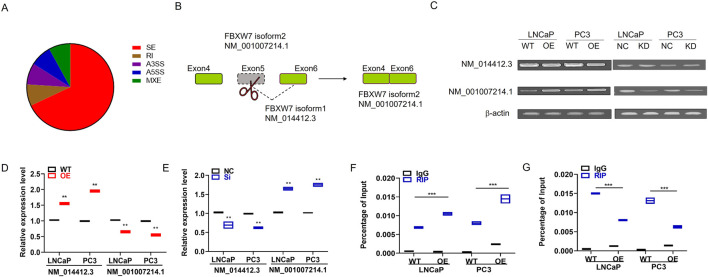
IMP3 regulates alternative splicing events in prostate cancer cells, including exon skipping of FBXW7. **(A)** RIP-seq analysis using HA antibody in IMP3-overexpressing prostate cancer cells identified 1,156 significant alternative splicing (AS) events, with exon skipping accounting for the majority (67.46%). **(B)** Among these, exon 5 skipping of the FBXW7 gene was one of the most prominently altered events in IMP3-OE cells. **(C–E)** Quantitative PCR showed that the expression of the alternatively spliced isoform FBXW7-NM_001007214.1 was significantly upregulated in LNCaP and PC3 cells overexpressing IMP3, while its expression was markedly decreased in IMP3-knockdown cells compared to controls. **(F,G)** RIP-PCR using an HA-tag antibody confirmed the direct binding of IMP3 to FBXW7-NM_001007214.1. Elevated IMP3 expression promoted the production of this isoform, supporting a regulatory role of IMP3 in exon skipping of FBXW7.

Given that IMP3 contains RNA-binding domains, including an RGG box and RNA recognition motifs—features commonly associated with RNA-binding activity. We conducted RNA immunoprecipitation sequencing (RIP-seq) using an HA-tag antibody to identify potential targets. This analysis revealed a total of 1,156 significantly altered alternative splicing (AS) events, with exon skipping being the most prevalent type, accounting for 67.46% of the events (Figure 3A).

To further elucidate how IMP3 influences alternative splicing (AS), we conducted a *de novo* motif analysis based on the overlapping exon sequences derived from IMP3-regulated AS events and IMP3-associated transcripts identified through RIP-seq. This analysis aimed to uncover potential RNA-binding motifs involved in IMP3-mediated regulation. Among the identified AS events, exon 5 skipping of the FBXW7 transcript was notably upregulated in IMP3-overexpressing cells and emerged as one of the most prominently affected splicing events (Figure 3B). We detected the expressions of the two isoforms FBXW7-NM_014412.3 and FBXW7-NM_001007214.1 in IMP3-OE cells by qPCR. Interestingly, the expression of FBXW7-NM_001007214.1 was increased in IMP3-OE cells relative to WT cells in both LNCaP and PC3 cells (Figures 3C–E). In comparison, the expression of FBXW7-NM_001007214.1 was significantly decreased in IMP3-KD cells compared with NC cells (Figures 3C–E). Furthermore, we adopted RIP-PCR to confirm IMP3-regulated exon skipping in PC cells using HA antibody as bait. It was found that IMP3 directly bound to the endogenous FBXW7-NM_001007214.1, and elevated IMP3 increased FBXW7-NM_001007214.1 expression compared with WT cells (Figures 3F,G). Collectively, we inferred that IMP3 regulated FBXW7 exon skipping, thereby spliced NM_014412.3 into NM_001007214.1, suggesting that IMP3-regulated FBXW7 exon skipping might play an important role in promoting PC progression.

### 3.4 Aberrant splicing of FBXW7 contributes to the reduction of c-myc ubiquitin

**FIGURE 4:**
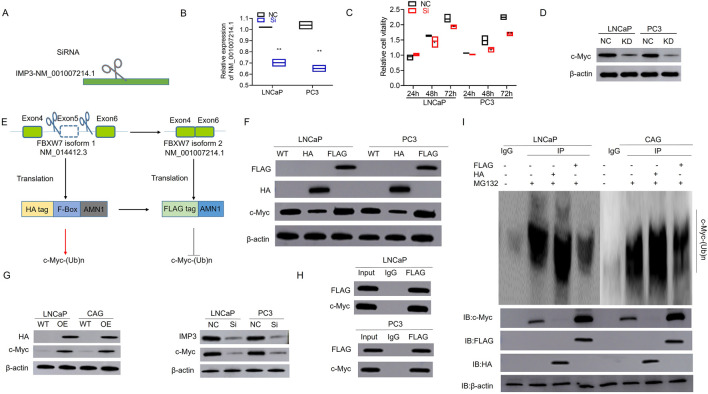
Aberrant splicing of FBXW7 impairs c-Myc ubiquitination and promotes its stabilization in prostate cancer. **(A,B)** A siRNA targeting the alternatively spliced FBXW7 transcript (NM_001007214.1) was designed and validated. **(C)** Silencing FBXW7-NM_001007214.1 significantly inhibited cell proliferation compared to negative control siRNA. **(D)** Western blot analysis showed decreased c-Myc protein levels in cells transfected with si-FBXW7 (NM_001007214.1), suggesting this isoform contributes to c-Myc stabilization. **(E)** Schematic representation of overexpression constructs encoding FBXW7-NM_014412.3 (HA-tagged) and FBXW7-NM_001007214.1 (FLAG-tagged), the latter lacking the F-box domain required for E3 ligase function. **(F)** Overexpression of FBXW7-NM_001007214.1 led to increased c-Myc protein levels, while FBXW7-NM_014412.3 overexpression suppressed c-Myc expression. **(G)** c-Myc expression was elevated in IMP3-overexpressing cells and reduced in IMP3-knockdown cells. **(H)** Co-immunoprecipitation confirmed a physical interaction between FBXW7 and c-Myc in prostate cancer cells. **(I)** After MG132 treatment (20 μM, 12 h), c-Myc ubiquitination was significantly reduced in cells overexpressing FBXW7-NM_001007214.1 compared to those expressing the canonical FBXW7 isoform (NM_014412.3).

To further investigate the functional impact of IMP3-regulated FBXW7 exon skipping and to elucidate the distinct roles of its two splicing isoforms in PC, we designed a siRNA specifically targeting FBXW7 transcript NM_001007214.1 (Figures 4A,B). As shown in Figure 4C, silencing this isoform significantly suppressed cell proliferation compared to the negative control. Given that FBXW7 is part of the SCF complex involved in c-Myc proteasomal degradation (Li S. R. et al., 2024), Western blot analysis revealed a marked reduction in c-Myc protein levels in cells transfected with si-FBXW7 NM_001007214.1 (Figure 4D). Notably, the protein encoded by FBXW7-NM_001007214.1 lacks the F-box domain present in FBXW7-NM_014412.3, which is required for SCF (SKP1-CUL1-F-box protein) E3 ubiquitin ligase function that mediates c-Myc ubiquitination and degradation (Boretto et al., 2024). To assess the biological roles of these two isoforms, we constructed HA- and FLAG-tagged overexpression plasmids for FBXW7-NM_014412.3 and FBXW7-NM_001007214.1, respectively (Figure 4E). Interestingly, overexpression of the FLAG-tagged NM_001007214.1 isoform led to increased c-Myc levels, whereas the HA-tagged NM_014412.3 isoform suppressed c-Myc expression (Figure 4F). Consistently, c-Myc expression was elevated in IMP3-overexpressing cells and reduced in IMP3 knockdown cells compared to controls (Figure 4G). Co-immunoprecipitation further confirmed the interaction between FBXW7 and c-Myc in PC cells (Figure 4H). Following treatment with the proteasome inhibitor MG132 (20 μM for 12 h), ubiquitination of c-Myc was notably diminished in FBXW7-NM_001007214.1-OE cells relative to those overexpressing FBXW7-NM_014412.3 (Figure 4I). These findings suggest that IMP3 regulates FBXW7 alternative splicing to favor production of the NM_001007214.1 isoform, which competes with the canonical isoform to impair c-Myc ubiquitination and promote its stabilization (Figures 4H,I).

### 3.5 circIMP3_288aa is identified as a circular RNA with its protein coding ability

**FIGURE 5:**
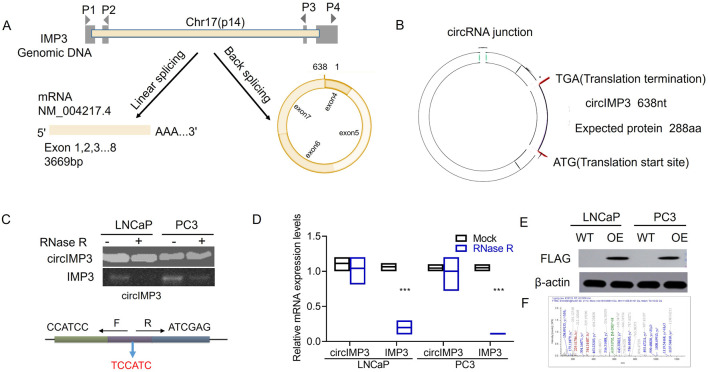
Diagram illustrating circIMP3 formation and its expression in prostate cancer. **(A)** Shows linear and back splicing of IMP3 genomic DNA. **(B)** Details circIMP3 circRNA with protein translation sites. **(C)** Gel image of circIMP3 presence in LNCaP and PC3 cells. **(D)** Bar graph comparing mRNA expression levels with and without RNase R treatment. **(E)** Western blots showing FLAG and β-actin expression in LNCaP and PC3 cells. **(F)** Mass spectrometry data of peptide analyses.

The tumor microenvironment plays a crucial role in supporting the oncogenic growth of PC cells. Recent investigations have begun to examine how circular RNAs (circRNAs) influence this microenvironment. Using the circBase database, we identified a 638 bp circular RNA derived from the IMP3 gene, predicted to be secreted and composed of 4 exons (Figure 5A). This circRNA harbors a putative internal ribosome entry site (IRES), suggesting its potential to encode a novel 288-amino-acid isoform of IMP3, hereafter referred to as circIMP3_288aa (Figure 5B). To verify the formation of this circRNA from exons 4 to 7, we designed convergent primers to detect linear IMP3 mRNA and divergent primers for the circular form. Following RNase R treatment, the linear transcript was significantly degraded (p < 0.001), while circIMP3 remained stable, indicating its circular nature (Figures 5C,D). Sanger sequencing further confirmed the back-splice junction characteristic of circIMP3 (Figure 5C). Using a FLAG antibody, we detected circIMP3_288aa protein in LNCaP and PC3 cells overexpressing IMP3 (Figure 5E). Mass spectrometry analysis corroborated the presence of unique peptide fragments corresponding to circIMP3-288aa (Figure 5F), confirming its protein-coding capacity. To evaluate its clinical relevance, we analyzed peripheral blood samples from 48 MM patients and 48 healthy donors. CircIMP3 expression was significantly higher in PC patients (*P* < 0.001; Figure 5G), and elevated expression correlated with poorer event-free survival (EFS) (*P* < 0.01; Figure 5H), suggesting that circIMP3 may serve as a potential biomarker for PC progression.

### 3.6 PC cells secrete circIMP3 into the BM microenvironment through exosomes

**FIGURE 6:**
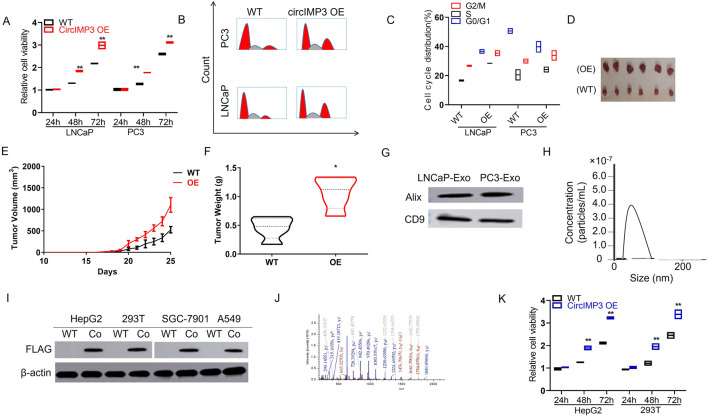
Prostate cancer cells secrete circIMP3 via exosomes to promote tumor growth and intercellular communication in the tumor microenvironment. **(A)** Overexpression of circIMP3 significantly enhanced proliferation of LNCaP and PC3 cells (p < 0.05). **(B,C)** Cell cycle analysis showed an increased proportion of cells in the G2/M phase upon circIMP3 overexpression. **(D–F)** *In vivo*, tumors derived from circIMP3-overexpressing LNCaP cells grew faster and exhibited significantly higher tumor weight and volume compared to wild-type controls (p < 0.05). **(G)** Western blot confirmed exosomal markers Alix and CD9 in exosomes isolated from LNCaP and PC3 culture supernatants. **(H)** Dynamic light scattering (DLS) analysis revealed exosome particle size averaging ∼50 nm. **(I)** FLAG immunostaining detected circIMP3_288aa protein in wild-type HepG2, 293T, RAW264.7, and A549 cells co-cultured with circIMP3-overexpressing Eca109 cells in a transwell system. **(J)** Mass spectrometry analysis confirmed the presence of circIMP3_288aa-derived peptides in recipient cells, indicating functional transfer and translation. **(K)** Co-cultured HepG2 and 293T cells exhibited significantly increased proliferation compared to non-co-cultured controls (p < 0.01).

**FIGURE 7:**
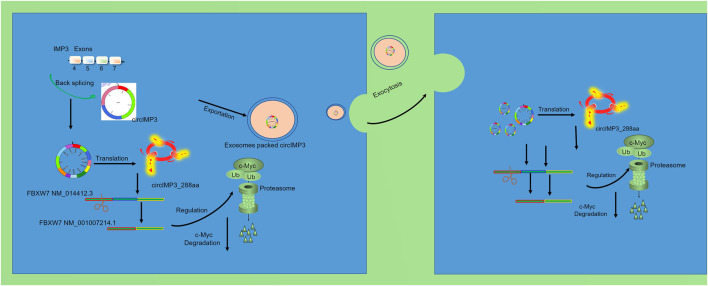
circIMP3 promotes prostate cancer progression through c-Myc stabilization and exosomal paracrine signaling.

To investigate the functional role of circIMP3_288aa, we constructed a FLAG-tagged plasmid encoding the full-length circIMP3 sequence. As shown in Figure 6A, overexpression of circIMP3 significantly promoted the proliferation of LNCaP and PC3 cells (p < 0.05). Cell cycle analysis further revealed a marked increase in the G2/M phase population in cells overexpressing circIMP3 (Figures 6B,C). To further validate the effect of circIMP3_288aa on PC proliferation *in vivo*, LNCaP WT and circIMP3-OE cells were injected subcutaneously into the right or left flanks of NOD-SCID mice, respectively. Tumors formed by circIMP3-OE cells grew more rapidly than those formed by WT cells, with significantly increased tumor weight and volume (p < 0.05) (Figures 6D–F). Given the critical role of the TME in PC progression, and the emerging understanding that circRNAs mediate intercellular communication, we examined whether circIMP3 could be secreted via exosomes. Exosomes isolated from the supernatants of LNCaP and PC3 cells were confirmed by the presence of canonical markers Alix and CD9 (Figure 6G). Dynamic light scattering (DLS) indicated that the exosomes had an average size of approximately 50 nm (Figure 6H). To assess the intercellular transmission of circIMP3_288aa, we co-cultured Eca109 circIMP3-overexpressing cells with wild-type HepG2, 293T, RAW264.7, and A549 cells using a transwell system. FLAG immunostaining revealed that circIMP3_288aa was detectable in all recipient cells (Figure 6I). Mass spectrometry further validated the presence of specific peptide fragments derived from circIMP3_288aa (Figure 6J), confirming the functional delivery and translation of circIMP3 in recipient cells. As depicted in Figure 6K, the proliferation rate of cocultured HepG2 and 293T cells was significantly increased (p < 0.01) relative to non-cocultured WT cells. In conclusion, our results demonstrate that PC cells can secrete circIMP3 via exosomes, enabling its translation into circIMP3_288aa in recipient cells and facilitating intercellular communication within the TME. In summary, this study identifies circIMP3, a highly upregulated circRNA in PC that encodes the oncogenic peptide circIMP3_288aa, drives FBXW7 exon skipping to stabilize c-Myc, promotes tumor progression both intracellularly and via exosome-mediated paracrine signaling, and serves as a promising prognostic biomarker detectable in blood (Figure 7).

## 4 Discussion

Prostate cancer (PC) remains a significant clinical challenge due to its high incidence, increasing mortality, and limited curative treatment options, particularly in advanced stages. While alternative splicing (AS) has been extensively studied as a key driver of PC progression, recent studies have highlighted the critical role of the tumor microenvironment (TME) and non-coding RNAs in regulating tumor behavior and therapeutic resistance (Marangon and Lecca, 2023; Sciarrillo et al., 2020).

Recent studies have highlighted AS as a fundamental post-transcriptional mechanism that affects the vast majority of eukaryotic genes and plays a pivotal role in cellular differentiation (Sciarrillo et al., 2020). Importantly, aberrant AS has been closely linked to tumor initiation and progression. During cancer development, alternative splicing generates diverse mRNA isoforms that give rise to structurally and functionally distinct proteins, contributing significantly to proteomic complexity (Tao et al., 2024; Bonnal et al., 2020). In malignant cells, the normal regulatory machinery of AS is frequently disrupted, resulting in cancer-specific transcriptomes that drive proliferation, metastasis, and resistance to therapy. Members of the IMP3 (IGF2BP3,Insulin-like growth factor 2 mRNA-binding protein 3) family, including IMP1 and IMP2, are known key modulators of AS. In our study, RIP-seq analysis revealed 2036 alternative splicing events regulated by IMP3, with a predominant enrichment in the ubiquitin-mediated proteolysis pathway. Further investigation demonstrated that IMP3 modulates exon skipping in the FBXW7 gene, thereby inhibiting c-Myc ubiquitination and promoting its stabilization. The ubiquitin-proteasome system (UPS) is essential for maintaining cellular homeostasis by controlling the degradation of key proteins involved in cell cycle regulation, apoptosis, gene expression, and proliferation—processes that are highly relevant to PC pathophysiology (Han et al., 2023). Clinically, patients with c-Myc translocations exhibit poorer progression-free survival (PFS) and overall survival (OS) (Gao et al., 2023).

In this context, circular RNAs (circRNAs), owing to their high stability, abundance in extracellular vesicles, and ability to encode functional peptides, have emerged as novel players in cancer biology. In the present study, we identified circIMP3, a circular RNA derived from the IMP3 gene, as being significantly upregulated in prostate cancer tissues and patient blood samples. Notably, we demonstrated that circIMP3 possesses protein-coding potential, generating a previously uncharacterized peptide of 288 amino acids, which we termed circIMP3_288aa. This finding contributes to the growing body of evidence that certain circRNAs are not merely non-coding regulators but may serve as templates for functional peptides with oncogenic properties. Functionally, overexpression of circIMP3_288aa significantly promoted the proliferation of prostate cancer cell lines (LNCaP and PC3) both *in vitro* and in xenograft mouse models. Mechanistically, circIMP3_288aa was found to modulate alternative splicing of FBXW7, a well-known tumor suppressor, suggesting that circIMP3 may drive tumor progression by altering post-transcriptional regulatory networks. This adds a novel layer of complexity to the oncogenic role of circRNAs, particularly those that function through translated products rather than via miRNA sponging or RNA-binding protein interactions alone.

An important and novel aspect of this study is the demonstration that circIMP3 is actively packaged into exosomes and secreted into the TME, where it can be taken up by various recipient cells, including epithelial and immune-derived cell lines (Mao et al., 2021; He et al., 2022; Yang et al., 2020). The internalization and translation of circIMP3_288aa in recipient cells led to enhanced proliferation, underscoring its potential role in mediating paracrine oncogenic signaling. These findings provide compelling evidence that circIMP3 not only functions cell-autonomously but also contributes to shaping a pro-tumorigenic microenvironment through intercellular communication. Clinically, circIMP3 expression correlated with poorer event-free survival, supporting its potential utility as a prognostic biomarker. The fact that circIMP3 is detectable in peripheral blood further reinforces its value as a minimally invasive liquid biopsy marker for PC diagnosis or disease monitoring.

In summary, this study uncovers a novel protein-coding circRNA, circIMP3, that promotes prostate cancer proliferation and modulates the tumor microenvironment through exosomal transfer. By elucidating the dual functional roles of circIMP3—in both intracellular signaling and extracellular communication—our findings provide new insights into the molecular underpinnings of PC progression and open up new avenues for therapeutic intervention.

## Data Availability

The original contributions presented in the study are included in the article/Supplementary Material, further inquiries can be directed to the corresponding authors.
