# Supplementary material for: A novel protein encoded by circIMP3 promotes prostate cancer progression by regulating alternative splicing and tumor microenvironment
Source: Front Cell Dev Biol. 2026 Jan 5;13:1722674. doi: 10.3389/fcell.2025.1722674 (PMC12813176; doi:10.3389/fcell.2025.1722674)
Supplement: Supplementary file 1 [file DataSheet1.pdf]

Nanopore Long-Reads RNA-seq:

A total of 1 μg of total RNA was used for cDNA library preparation following the standard protocols provided by Oxford Nanopore Technologies (ONT). Full-length mRNAs were reverse-transcribed using the SuperScript IV First-Strand Synthesis System (Invitrogen) and subsequently amplified for 14 PCR cycles with LongAmp Taq (NEB). The amplified products underwent FFPE DNA repair and end-repair (NEB), followed by adapter ligation using T4 DNA ligase (NEB). DNA purification was performed with Agencourt XP beads according to the ONT protocol. The final cDNA libraries were sequenced on the PromethION platform (Biomarker Technology, Beijing, China). Raw reads were filtered using a minimum average quality score ≥ 7 and minimum read length ≥ 500 bp, and rRNA-derived sequences were removed. High-quality full-length, non-chimeric transcripts were obtained by mapping reads to the hg38 reference genome using minimap2 (v2.18), followed by collapsing redundant isoforms using the cDNA Cupcake package. After polishing with pinfish, consensus transcript isoforms were generated.

We first aligned RIP-seq reads to the human reference genome using STAR with parameters optimized for spliced alignments. AS events enriched in IMP3-bound RNA were identified using rMATS (v4.1.0), which quantifies five major types of splicing events. Significant AS events were defined as $P < 0.05$ and |IncLevelDifference| > 0.05. To complement RIP-seq findings and validate full-length splice isoforms, we further analyzed Nanopore long-read sequencing data. We believe these clarifications clearly distinguish our RIP-seq–based enrichment analysis from conventional RNA-seq–based AS profiling and demonstrate the validity of our approach.

We analyzed IMP3-regulated AS events in IMP3-knockout and control LNCaP cells using rMATS for RIP-seq data and FLAIR for Nanopore long-read RNA-seq data. The two algorithms identified 1,156 and 1,153 AS events,

respectively (Figs. 1A-B). Examination of the FBXW7 locus in the Nanopore long-read dataset further revealed that IMP3 knockdown enhanced the retention of exon 5, as illustrated by the corresponding Sashimi plot (Fig.1C).

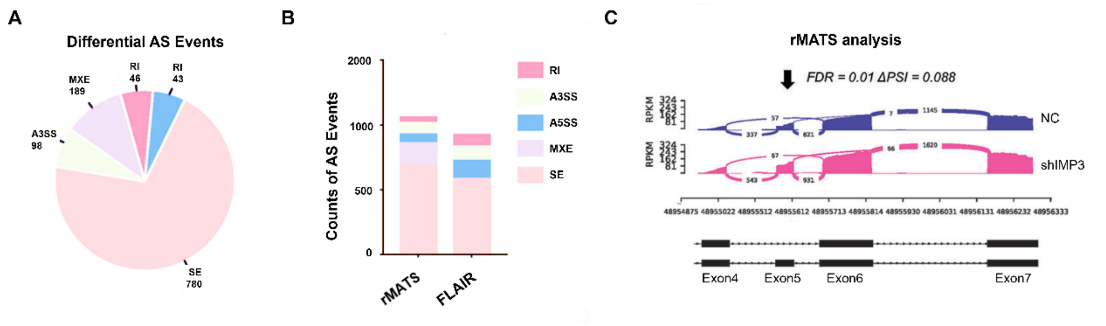

Figure 1 IMP3-mediated alternative splicing events. (A) Pie chart showing the distribution of significantly altered AS events identified after IMP3 knockdown, analyzed and filtered using rMATS (P < 0.05, |IncLevelDifference| > 0.05). SE, skipped exon; RI, retained intron; A5SS, alternative 5′ splice site; A3SS, alternative 3′ splice site; MXE, mutually exclusive exons. (B) Numbers of different types of AS events detected using rMATS based on RIP-seq data and FLAIR based on Nanopore long-read RNA-seq data. (C) Sashimi plot analysis of Nanopore Long-Reads RNA-seq data showing the FBXW7 AS event after IMP3 knockdown in LNCaP cells, with the PSI value for exon 5 skipping calculated using MISO.
